# Cost minimization analysis of digital-first healthcare pathways in primary care

**DOI:** 10.1038/s41746-025-01937-z

**Published:** 2025-08-25

**Authors:** Alexandra Dahlberg, Sakari Jukarainen, Taavi Kaartinen, Petja Orre

**Affiliations:** 1 Harjun terveys, Helsinki, Finland; 2 Faculty of Medicine, University of Helsinki, Helsinki, Finland; 3 Mehiläinen, Helsinki, Finland

**Keywords:** Health care economics, Health services, Public health

## Abstract

A retrospective, registry-based cost-minimization analysis assessing whether initiating minor acute primary care episodes via a digital-first pathway reduces costs compared to traditional care in a Finnish setting. Of 637,923 encounters, 64,969 eligible episodes were identified in five selected clinical presentations. After propensity score matching (19,697 pairs), mean episode costs were significantly lower in the digital-first pathway (€170.74) than in traditional care (€220.91), reflecting a 22.7% reduction (*P* < 0.001). Savings varied by clinical presentation, from 10.3% for respiratory infections to 52.5% for gastroenteritis (all *P* < 0.001). Digital care was associated with lower use of laboratory tests and imaging. Follow-up visits were generally fewer in the digital-first pathway, except for respiratory infections, which showed a slight increase. Sensitivity analyses with 7- and 30-day follow-up windows produced similar results. Overall, this study supports digital-first models as a cost-effective strategy for managing minor acute conditions in primary care.

## Introduction

Healthcare systems globally face growing financial pressures due to aging populations^1^, and increasing prevalence of chronic diseases. This underscores an urgent need for innovations that enhance efficiency and cost-effectiveness in care delivery. Digital health technologies – including telemedicine, mobile health applications, and online patient portals – have emerged as promising solutions to deliver primary care more efficiently^2–4^. By reducing overhead, optimizing clinician time, and streamlining patient management, digital interventions have the potential to decrease overall healthcare expenditures. Systematic reviews have indicated that many digital health interventions can be cost-effective and sometimes even cost-saving^5^. However, real-world evidence demonstrating economic benefits in routine healthcare settings remains limited^6^.

One analytical approach for comparing costs in such settings is cost-minimization analysis, which is appropriate when clinical outcomes of the interventions are expected to be equivalent^7^. In the context of acute, minor health issues (for example, managing an uncomplicated infection via a digital visit versus an office visit), the health outcomes are generally assumed to be comparable. Thus, any difference in cost between a digital-first and a traditional pathway would primarily reflect differences in efficiency. Focusing on cases where outcomes are likely equivalent allows us to directly evaluate whether digital delivery economizes resources compared to traditional care. However, confounding factors may influence the analysis, as patients choosing digital care may differ in demographic or clinical characteristics^8–10^. To address this, propensity score matching (PSM) was applied to account for these factors.

This study examines the cost-efficiency of digital healthcare implemented in a large-scale primary care setting in Finland. In the Päijät-Häme Wellbeing Services County in Finland, patients with acute primary care needs can seek care either by using a digital clinic app (Päijät-Sote) or through traditional means like calling a health center or walking in. This parallel system enables a direct comparison of care pathways across common minor acute conditions, including dermatological symptoms, gastroenteritis, ophthalmologic symptoms, respiratory infections, and urinary tract infections. While a single digital encounter is generally associated with lower overhead costs compared to traditional in-person care, failure demand (e.g., low value first digital contact leading to an immediate in-person visit) might lead to higher total costs. Thus, we examine costs for up to 14 days from the first contact (episode costs), striving to quantify the total economic impact of digital-first care for the selected clinical presentations. This allows us to assess whether digital care serves as cost-efficient substitutes or function merely as additional low value steps in the care process for these conditions.

## Results

### Study population

After applying inclusion criteria and propensity score matching (PSM), the final study population consisted of 19,697 matched episode pairs. Each matched pair comprised of one episode initiated digitally and one initiated traditionally (phone call or inpatient visit), matched on patient characteristics and diagnostic category. The matched episode pairs were distributed as follows: 6927 dermatologic symptoms, 312 gastroenteritis, 1461 ophthalmologic symptoms, 9616 respiratory infections, and 1381 urinary tract infections (Table 1). Before matching, patients receiving care via the digital-first pathway were consistently younger across all diagnostic groups. Healthcare utilization metrics also showed clear differences before matching (Table 2). The traditional pathway exhibited higher mean encounters, laboratory tests, and imaging procedures in most diagnostic categories compared to the digital-first pathway. After matching, mean encounter counts were closely aligned – 2.23 for digital-first and 2.28 for traditional episodes.

**Table 1:** Characteristics of patients in each care pathway before and after propensity score matching (PSM)

	Unmatched			Matched		
Pathway	Age, mean	Female (%)	Number of episodes	Age, mean	Female (%)	Number of episodes
Dermatological symptoms						
Digital	29.5	62.9	7890	31.7	60.3	6927
Traditional	56.8	57.1	19,008	32.7	60.9	6927
Gastroenteritis						
Digital	23.4	61.1	804	28.6	63.1	312
Traditional	37.6	57.0	393	28.8	57.4	312
Ophthalmological symptoms						
Digital	24.9	63.9	3149	35.0	62.6	1461
Traditional	51.1	64.9	2454	36.2	63.0	1461
Respiratory infections						
Digital	25.6	63.0	9616	25.6	63.0	9616
Traditional	36.8	60.9	17,958	25.7	63.0	9616
Urinary tract infections						
Digital	33.3	92.1	1933	35.4	89.6	1381
Traditional	39.8	84.4	1764	35.0	89.1	1381

Mean age, proportion of female patients, and number of episodes are shown for each clinical presentation and pathway (digital-first vs. traditional), both before (unmatched) and after PSM. Matching was performed separately within each diagnostic category to ensure balance between pathways.

**Table 2:** Healthcare utilization by pathway before and after propensity score matching (PSM)

	Unmatched			Matched		
Pathway	Number of encounters (mean per episode)	Number of labs (mean per episode)	Number of imaging (mean per 100 episode)	Number of encounters (mean per episode)	Number of labs (mean per episode)	Number of imaging (mean per 100 episode)
Dermatological symptoms						
Digital	1.98	0.90	1.38	2.00	0.96	1.53
Traditional	2.41	1.91	4.75	2.23	1.51	3.39
Gastroenteritis						
Digital	1.85	0.86	0.62	1.78	0.98	0.64
Traditional	2.87	6.05	4.33	2.72	4.38	3.85
Ophthalmological symptoms						
Digital	2.18	0.36	0.51	2.26	0.56	0.82
Traditional	2.49	1.56	3.91	2.46	1.17	3.70
Respiratory infections						
Digital	2.34	1.00	2.24	2.34	1.00	2.24
Traditional	2.32	1.97	7.87	2.18	1.54	4.35
Urinary tract infections						
Digital	2.59	4.72	1.04	2.62	4.90	1.16
Traditional	2.98	8.62	3.40	2.89	8.41	3.11

The table presents the mean number of encounters and laboratory tests per episode, and imaging procedures per 100 episodes, for each clinical presentation and care pathway (digital-first vs. traditional), both before (unmatched) and after PSM. The matching was performed separately by diagnostic category.

### Cost comparison

Overall, episodes managed via the digital-first pathway incurred significantly lower costs compared to those in the traditional pathway, with a mean cost of €170.7 vs. €220.9, representing a 22.7% cost reduction (*P* < 0.001). This trend was consistently observed across the dataset (Table 3). On average, digital-first episodes were €50.17 less expensive per episode (*P* < 0.001) than those initiated through traditional care.

**Table 3:** Mean cost per episode by healthcare pathway (digital-first vs. traditional)

Category	Digital-first pathway (€)	Traditional pathway (€)	*P*-value	Cost savings
Encounter costs, mean per episode	166.54	212.68	<0.001	21.7%
Laboratory costs, mean per episode	3.29	6.21	<0.001	47.0%
Imaging costs, mean per episode	0.91	2.01	<0.001	54.7%
Total, mean per episode	170.74	220.91	<0.001	22.7%

Mean costs per episode are presented for encounters, laboratory tests, imaging, and total episode cost. Cost comparisons between digital-first and traditional pathways were conducted using independent two-sample *t*-tests. *P*-values reflect statistical significance. Percentage cost savings represent the relative reduction in the digital-first pathway, calculated as (Traditional – Digital-first)/Traditional.

**Fig. 1:**
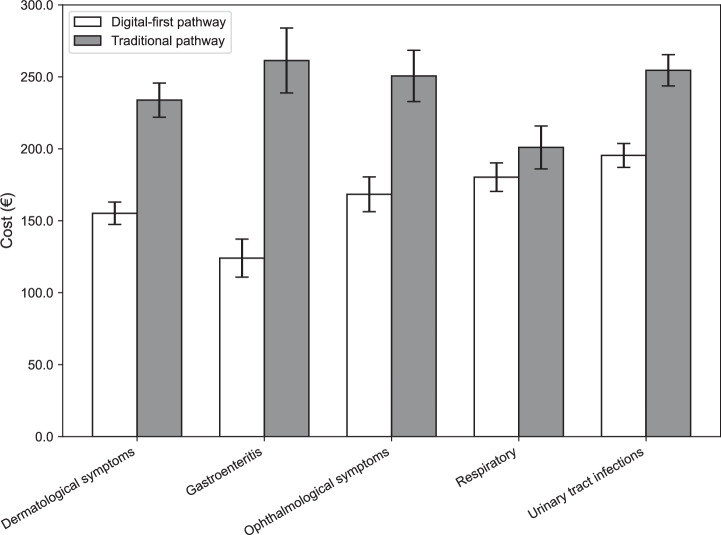
Mean episode costs by clinical presentation in digital-first vs. traditional pathways. Bar chart comparing the mean total healthcare cost (€) per episode across five minor acute clinical presentations in primary care, with error bars indicating 95% confidence intervals.

Table 4 and Fig. 1 details the cost outcomes by clinical presentation. In every category, the mean episode cost in the digital-first pathway was significantly lower than in the traditional pathway (*P* < 0.001 for each).

**Table 4:** Mean total cost per episode by clinical presentation and healthcare pathway

	Digital-first pathway, mean per episode (€)	Traditional pathway, mean per episode (€)	Cost savings	*t*-statistic	*P*-value	N of pairs
Dermatological symptoms	155.21	233.81	33.6%	−29.72	<0.001	6927
Gastroenteritis	124.02	261.36	52.5%	−10.34	<0.001	312
Ophthalmological symptoms	168.38	250.61	32.8%	−13.40	<0.001	1461
Respiratory infections	180.27	200.96	10.3%	−9.20	<0.001	9616
Urinary tract infections	195.38	254.51	23.2%	−9.20	<0.001	1381

Mean episode costs (in euros) are shown for digital-first and traditional pathways across clinical presentation categories. Cost savings represent the relative difference, calculated as (Traditional – Digital-first)/Traditional. Independent two-sample *t*-tests were used for comparisons, with *t*-statistics and *P*-values reported. Sample sizes reflect the number of matched episode pairs.

Across conditions, the gastroenteritis group showed the largest relative cost saving: digital-first gastroenteritis episodes cost 52.5% less than traditional episodes (€124.02 vs. €261.36). This was followed by dermatologic symptoms with 33.6% savings (€155.21 vs. €233.81) and ophthalmologic symptoms with 32.8% savings (€168.38 vs. €250.61). The urinary tract infections group saw about 23.2% lower costs with digital initiation (€195.38 vs. €254.51). The respiratory infection group, which was the largest sample size, had a more modest 10.3% cost reduction (€180.27 vs. €200.96) via digital-first pathways.

Looking at cost components, we found that the cost gap was primarily driven by differences in encounter costs. While laboratory and imaging utilization was generally low for these minor acute conditions, we observed statistically significant differences, with consistently lower test use in the digital-first group. For example, in urinary tract infections, clinicians in the traditional pathway more frequently order confirmatory laboratory tests and urine cultures. Overall, laboratory costs averaged lower in digital-first episodes (€3.29 vs €6.21, a 47.0% saving). Imaging usage was minimal for these diagnoses, with associated costs roughly 50% lower for digital-first episodes (mean €0.91 vs €2.01). Cost breakdowns by diagnostic categories and the modality of follow-up encounters are provided in the supplementary attachment (Supplementary Table 1, Supplementary Figs. 1 and 2).

### Sensitivity analyses

The following sensitivity analyses were performed: (a) varying the episode follow-up window to 7 and 30 (b) conducting an analysis on unmatched data utilizing multivariable regression; (c) adjusting cost assumptions to assess the influence of different pricing structures on the overall findings^11,12^; and (d) employing inverse probability of treatment weighting (IPTW) with truncated weights at the 1st and 99th percentile^13–15^. Results from sensitivity analyses did not meaningfully affect the results or conclusions (Supplementary Tables 2–8).

## Discussion

In our study setting common minor acute primary care episodes initiated via a digital-first pathway were associated with approximately 23% lower costs on average compared to traditional pathways, without increasing follow-up visits or diagnostic test usage. By analyzing 17 months of data from a Finnish primary care setting, we observed consistent cost reductions across multiple diagnostic categories. These findings reinforce prior research suggesting that digital health interventions could potentially generate economic benefits^16^.

The observed cost-effectiveness of the digital-first pathway is primarily driven by lower encounter costs. Chat-based digital care has the potential to reduce costs primarily through efficiency gains from the use of automated and standardized questionnaires and asynchronous communication (the same healthcare professional can evaluate multiple patients simultaneously). Our findings suggest that digital consultations are often sufficient for managing the examined conditions, with no observed increase in subsequent healthcare contacts compared to traditional pathways. Consequently, encounter costs in the digital-first pathway are substantially lower and account for the majority of observed cost savings (Table 3). Differences in laboratory testing and imaging utilization were present but contributed less significantly, underscoring that encounter costs are the primary driver of savings.

Moreover, our results suggest that fewer laboratory and imaging studies are ordered in digital-first pathways. Conservative testing practices are encouraged in the examined digital care model and management of common issues is more heavily standardized. Healthcare professionals may emphasize clinical history and adopt a watchful waiting approach for mild cases. In a traditional in-person setting clinicians may have a tendency to perform additional diagnostic tests as a precaution or to reassure the patient. Overuse of low-value diagnostics in traditional settings can contribute to increased healthcare expenditures, whereas digital health platforms may inherently promote more judicious resource utilization via stronger standardization of practices.

These findings contribute to the growing body of evidence on the effectiveness of digital consultations in primary care. Glock et al. demonstrated that eVisits in Swedish settings were suitable for managing uncomplicated conditions, with the majority of patients not requiring follow-up^17^. Likewise, studies focusing on minor acute illnesses have generally reported no increase in downstream utilization following digital encounters^18–23^, with some reporting reduced service use^24^. However, earlier investigations have also noted increased follow-up visits and higher healthcare utilization following digital encounters^8,25–28^, particularly in cases of acute respiratory tract infections^29^. While few studies have assessed costs directly, those that have typically suggest favorable economic outcomes^5^. One exception is the study by Ashwood et al.^30^ which found higher costs for digital consultations in respiratory cases. Notably, respiratory infections represented the only diagnostic category in our study with increased follow-up rates and the smallest relative cost savings.

The magnitude of cost savings in our study, ranging from 20 to 50% depending on condition, is higher than previously reported in some settings. For example, Buvik et al. observed a 19% cost reduction in orthopedic teleconsultations, contingent upon sufficient patient volume^31^. Gentili et al. found that over half of the studies in their review reported digital interventions as dominant – both more effective and less costly^5^. Ayabakan et al. similarly reported a 13.6% reduction in future outpatient visits and $239 in cost savings within 30 days of a telehealth encounter, with the strongest effects observed in conditions amenable to virtualization, such as mental health, skin, metabolic and musculoskeletal diseases^32^. On the other hand, a scoping review in Australia cautioned that telehealth’s system-wide savings were not always realized because of how services were funded and organized^33^. Importantly, our study setting enabled digital consultations to function as true substitutes. The digital platform is integrated with clinical infrastructure, allowing for laboratory orders, referrals, and escalation to in-person visits when necessary. This integration is critical – if digital services merely add another layer of care, overall utilization and costs may increase. In our context, where the same provider organization managed both digital and traditional services, care was coordinated to avoid unnecessary steps or layers. In our study digital encounters did not result in excess follow-ups, reinforcing the role of digital care as a substitute rather than a supplement.

These results have meaningful implications for healthcare policy. Scaling digital-first care models, particularly for conditions shown to be safely manageable online, may offer a viable strategy for health systems seeking cost savings. Importantly, these savings do not require workforce reductions but could instead alleviate pressure to scale staffing proportionally with rising care demands – particularly as populations age and service needs increase. However, careful attention to clinical quality and health equity is warranted. Digital care protocols must include safeguards such as appropriate triage and escalation of care. Quality monitoring mechanisms are essential to ensure that cost reductions do not compromise clinical outcomes.

Another major implication is the need to address the digital divide. Our findings highlight a considerable age disparity in digital-first pathway utilization, with younger patients disproportionately using digital healthcare services. This aligns with prior evidence suggesting that older adults, individuals with limited internet access, and those with lower digital literacy are less likely to engage with digital health solutions^7,8^. Additional barriers may also affect individuals with cognitive impairments or language difficulties. If these disparities remain unaddressed, the benefits of digital healthcare – such as improved access, convenience, and cost savings – may be inequitably distributed, reinforcing existing healthcare inequalities.

However, digital health also has the potential to enhance accessibility, particularly in remote or underserved regions where traditional healthcare infrastructure is limited^34,35^. In the studied time period virtually all mobile phone plans in Finland include internet access, with full coverage of the geographical area. Additionally, patient fees for digital and traditional consultations are comparable, typically zero euros when only the initial contact is involved, suggesting that financial barriers are unlikely to explain differences in uptake. Nonetheless, disparities persist in digital engagement, as not all individuals are willing or able to use digital services. To ensure equitable adoption, targeted interventions should be implemented, including digital literacy programs tailored to older populations, and user-friendly app designs that accommodate a wide range of technological proficiencies. Moreover, successful integration of digital care could free up resources for patients preferring the traditional settings, potentially improving access outside digital care.

Our results also feed into the broader discussion of healthcare system reform. Finland’s healthcare reform seeks cost savings to rein in deficits, and digital health has been highlighted as a key strategy. The results from this study supports those strategic directions – a digital-first primary care model could indeed contribute to cost containment in the region. Policymakers and administrators can leverage these findings to justify investments in digital platforms. While there is an upfront cost to building and maintaining apps and IT infrastructure, the return on investment appears favorable given the per-episode savings. Additionally, while our analysis focused on the provider’s perspective, other benefits such as reduced transportation costs, lower absenteeism, patient preference^36–39^ and reduced emissions^40,41^ further strengthen the case for digital-first pathways, even though these were not quantified in this study.

This study has several limitations to acknowledge. First, despite propensity score matching, residual confounding may bias the estimates. Unmeasured factors – such as clinical complexity, digital literacy, patient preference or provider differences – may have influenced both care pathway selection and costs. As illustrated in Supplementary Figs. 1 and 2, patients appear to favor the same modality for follow-up visits, suggesting that patient preference may play a role. This may introduce behavioral confounding, such as self-selection into digital or in-person encounters. Furthermore, physicians in the digital clinic are compensated on an hourly basis, whereas their in-person counterparts receive fixed monthly salaries. This difference in payment structure could potentially influence clinical engagement and decision-making incentives. Additionally, some differences in prescribed medications remained between groups after matching, reflecting in part differences in case-mix and differences in clinical management (Supplementary Tables 9 and 10). Nonetheless, the observed cost differences were substantial, making it unlikely that residual confounding alone explains the findings.

Second, as a cost-minimization analysis, we did not directly assess clinical outcomes such as symptom resolution, patient satisfaction, or complication rates. We assumed equivalent outcomes between pathways based on clinical judgment and similar follow-up rates (Table 2). We did not attempt to assess differences in clinical outcomes due to limited statistical power, since outcomes reliably ascertainable via available registry data are rare (e.g. hospitalization due to pneumonia). While prior studies suggest that telemedicine provides comparable outcomes for common acute conditions, differences in quality or patient outcomes cannot be ruled out.

Third, our findings are limited to acute, low-complexity conditions commonly managed in primary care. This analysis therefore excludes inpatient encounters. This may lead to an underestimation of total costs in episodes involving hospital care, although such events are expected to be rare given the low-acuity nature of the conditions studied. Furthermore, results may not generalize to clinical presentations we did not study, for example management of chronic illnesses in multimorbid patients, mental health or primary prevention. Evaluating the cost and clinical performance of digital-first care for these populations remains an important area for future research.

Fourth, the study is region-specific, focusing on Päijät-Häme in Finland, within a healthcare system that integrates digital services into public primary care. The digital clinic provides quick access to care, with extensive opening hours, typically with a low waiting time of less than 15 minutes, and virtual clinicians have the same capabilities as phone-based services for scheduling appointments and issuing referrals. The region also maintains strong access to in-person care, with acute appointments frequently available on the same day. While the findings are likely generalizable to settings with similar infrastructure and care models, their applicability to healthcare systems with different payment structures – such as purely fee-for-service models – is uncertain. Differences in reimbursement incentives, physician practice patterns, patient digital literacy, and clinician acceptance may all influence care processes and outcomes, potentially limiting the transferability of our results to other healthcare environments.

Fifth, the time horizon was relatively short (14 days, with a 7-day and 30-day sensitivity analyses), capturing only the associated acute episode costs. While this timeframe is appropriate for evaluating short-term healthcare expenditures^17^, it does not account for any potential long-term cost implications. We chose a relatively short time horizon as the registry data does not allow separation of related and unrelated costs, consequently the longer the horizon, the more it is contaminated by unrelated costs. If digital consultations led to increased downstream healthcare utilization beyond the 30 days, such effects would not be captured in this analysis. Both the supplementary analysis of follow-up care (Supplementary Fig. 3) and the 30-day cost sensitivity analysis (Supplementary Table 6) suggest that cost savings observed for digital-first care were not offset by increased resource use later in the care pathway. Nonetheless, longer-term studies are warranted, particularly to assess the economic impact of digital-first pathways for chronic disease management.

Sixth, cost estimates are inherently subject to variability. Our analysis is based on publicly available regional cost data, which represents average production costs rather than real-time, transaction-level expenditures. While this approach aligns with economic evaluation best practices, healthcare cost structures vary across systems, and the results may not fully capture hidden or indirect costs associated with digital-first and traditional pathways.

Seventh, episode-based costing may include services unrelated to the minor acute condition. However, this applies equally to both groups and is unlikely to introduce systematic bias.

Eight, the study examined costs solely from the healthcare provider’s perspective, excluding societal costs such as patient travel expenses, lost work time, or productivity losses. Given that digital consultations eliminate the need for physical travel and reduce time burdens for patients, incorporating these factors in future cost-benefit analyses would likely strengthen the case for digital-first pathways.

In summary, this study supports the view that digital-first primary care pathways could reduce healthcare costs for the examined common minor acute conditions in primary care without a significant increase in follow-up visits. In the examined Finnish healthcare system, digital-first pathways lowered the cost of managing common infections and minor illnesses by approximately 20-50%, depending on the condition. These findings support the integration of digital health solutions as a scalable strategy to improve efficiency in healthcare systems with similar structures.

However, achieving widespread adoption while ensuring equitable access and maintaining clinical quality will be critical. With thoughtful implementation, digital-first pathways may help ease pressure on rising healthcare expenditures, a key priority for the long-term sustainability of health systems globally.

## Methods

### Study design and setting

We conducted a retrospective registry-based study with a cost-minimization analysis framework^42^. This approach was selected based on the premise that both digital-first and traditional care pathways manage the same minor acute conditions under standardized clinical guidelines, with similar outcomes for the patient. Assuming equivalent clinical effectiveness, cost-minimization analysis allows for a direct evaluation of economic efficiency by comparing healthcare expenditures between the two pathways.

The study was conducted in the Päijät-Häme region of Finland, within Harjun terveys a public-private joint venture that provides typical Finnish publicly funded all-inclusive primary healthcare services. The system is primarily financed through taxation, which ensures that care is low-cost at the point of use. In most cases, patients pay only a small fee per visit for primary care services. Harjun terveys serves the municipalities of Lahti, Iitti, Kärkölä, and Hartola, covering a population of approximately 134,000. Harjun terveys has increasingly adopted digital services in recent years. Since 2021, these have included digital primary care and mental health clinics, messaging with one’s care team, and access to personal health records. A dedicated digital primary care clinic (the Päijät-Sote digital platform) was introduced in this region on May 1, 2023, operating alongside traditional care. The study observation period ran from May 1, 2023 through September 30, 2024, covering the first 17 months of the digital clinic’s implementation.

This research followed the Consolidated Health Economic Evaluation Reporting Standards (CHEERS) guidelines (Supplementary Table 11)^43^. All necessary permissions for the study were obtained from the Päijät-Häme Wellbeing Services County. Data use complied with the Finnish Act on the Secondary Use of Health and Social Data^44^, and all applicable data protection regulations, including the EU General Data Protection Regulation (GDPR)^45^.

### Inclusion criteria

All patients with their home address in the catchment area and those who were alive for the study period were included. All primary care contacts with nurses and doctors were included, except contacts related to health promotion (e.g., vaccinations, maternity and child health clinics). Secondary, tertiary, and inpatient care were not examined. We defined an “episode” as a fixed period of 14 days following the initial index encounter. We restricted the analysis to five categories of clinical presentation: dermatological symptoms, gastroenteritis, ophthalmologic symptoms, respiratory infections and urinary tract infections. These diagnostic categories were chosen to represent typical minor acute conditions commonly managed in primary care and amenable to digital consultation (e.g., no definite need for a physical examination). In our dataset, these diagnostic categories accounted for 51.7% of digital encounters and 23.0% of traditional encounters (phone or walk-in), comprising 27.4% of all encounters in primary care after the exclusion of contacts related to health promotion. Episodes were included if the primary diagnosis at the index encounter matched one of the predefined categories, as identified by ICD-10 and ICPC-2 codes in the registry (Supplementary Table 12). Finally, for urinary tract infection episodes, we excluded patients over 65 years old; national clinical protocol does not recommend remote management of UTIs in this age group.

### Digital-first vs. traditional pathway definition

The digital-first pathway was defined as episodes where the patient initially contacts the healthcare professional through the Päijät-Sote digital platform. On the platform the patient, or guardian on behalf of the patient, initiates the digital encounter either by filling out an automated symptom questionnaire or by describing one’s symptoms with free-form text. A registered nurse then assesses the need for treatment by reviewing the completed questionnaire or the free-form text and by interviewing the patient via chat when necessary. Nurses consult a physician in the virtual team when needed. If warranted, the patients can be transferred to a chat with a digital physician, issued laboratory test orders or referred to another point of care – all delivered digitally within the same care continuum. The traditional pathway episodes began with either a phone appointment, an in-person appointment booked through a treatment needs assessment phone call or as walk-ins to a clinic. The digital and traditional services were both staffed by primary care nurses and physicians; however, while the traditional pathway was exclusively operated by local healthcare professionals, the digital platform is operated by a dedicated virtual team. Both teams consist of nurses and general practitioners with comparable levels of training and seniority, although the traditional care team is slightly more heterogeneous. The median age of nurses in the virtual team is closely aligned with that of the physical team (40 vs. 39), and their median seniority is nearly identical (11.17 vs. 11.08 years). Among doctors, most in the virtual team work on an hourly basis, so years of experience are not systematically recorded. The ratio of nurses to medical doctors is consistent across both pathways, and all providers adhere to national and locally defined protocols and guidelines to ensure uniformity and consistency in care delivery. Importantly, after the initial contact, patients in both pathways could receive additional care as needed, including follow-up visits (digital or traditional), tests, and referrals.

**Fig. 2:**
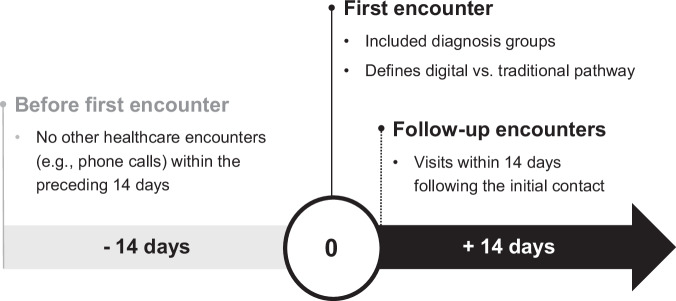
Conceptual definition of an episode of care. An episode begins with an index (first) encounter, provided there were no prior related contacts in the preceding 14 days, and includes any follow-up visits occurring within 14 days after the initial contact.

An episode was established when a patient first contacted healthcare services via either the digital-first or traditional pathway, requiring that no prior healthcare encounters had occurred within the preceding 14 days. Each episode’s utilization was then tracked for 14 days from the initial contact (Fig. 2). This 14-day follow-up window was selected based on the acute and self-limiting nature of the studied conditions, which typically resolve within one to two weeks. Moreover, comparable follow-up periods are frequently used in prior studies evaluating similar minor acute conditions, especially in digital health contexts^17–21,23,25–27,32,46^. The majority of the encounters occurred within the first two weeks after the initial encounter, with utilization declining substantially thereafter towards baseline levels (Supplementary Fig. 3). Our cost analysis encompassed all primary care services utilized within this 14-day follow-up window, irrespective of modality. The first ICD-10 or ICPC-2 code present during an episode was used to determine the clinical presentation-category, so for example if the first encounter had no recorded code the next encounter with a code was used.

### Cost estimation

We obtained cost data from the provider’s administrative database and regional cost catalogs. Each healthcare encounter in primary care has an associated cost based on standard tariffs (e.g. staff time and facility use, etc.), and each laboratory test and imaging has a unit cost. We used the Päijät-Häme Wellbeing Services County public standard pricing catalog for public primary care services to assign costs (Supplementary Table 13)^47^. These prices reflect the health system’s perspective (approximately the cost or tariff charged for providing the service). Costs were calculated in Euros (€) at 2024 price levels. For each episode, we summed: (1) all encounter costs (clinic visits, digital consultations, phone consultations), (2) any laboratory test costs ordered during the episode window, and (3) any imaging costs (e.g. if an X-ray was done). We did not include indirect costs (like patient travel expenses or lost productivity) in the base analysis – our perspective is that of the healthcare provider/payer.

### Outcome measures

The primary outcome was the difference in mean total episode cost between digital-first and traditional pathway episodes, for each diagnostic group and overall. We calculated mean cost per episode for each group in each pathway, and the percentage difference (cost saving). Secondary outcomes included the breakdown of cost components (encounter vs. laboratory vs. imaging) by pathway, and the rate of follow-up visits in 14 days (to assess if one pathway led to more revisits).

### Statistical analysis

To control confounding factors, we applied 1:1 propensity score matching (PSM) to match digital-first and traditional episodes^48–50^. Propensity scores were estimated with a logistic regression model, using age, sex, Charlson comorbidity index (CCI), and number of primary care doctors’ visits in the 2 years prior to the start of the observation period (May 1, 2023)^51^. Quadratic and cubic terms were included for age and for the prior visit count in the propensity model. The CCI, a measure of comorbidity burden that predicts mortality risk based on the presence and severity of chronic conditions^51^, was calculated excluding age, which was modeled separately. The CCI score was restricted to have a maximum of 3 due to heavy skewness. Episodes were matched within the types of clinical presentation.

Each digital-first episode was matched to one traditional episode with the closest propensity score (nearest-neighbor matching with caliper = 0.2)^52^, if a match within the caliper was available. Using the matched pairs of episodes, we compared costs between pathways with independent two-sample *t*-tests^53,54^. We report on means, *t*-statistics and *P*-values for the cost differences. A *P* < 0.05 (two-tailed) was considered statistically significant. CCI calculations were performed using an R package^55^, while other statistical analyses were conducted using Python software^56–60^.

The regional healthcare authority, Päijät-Häme Wellbeing Services County approved the study plan and data use agreement (decision number HA/85/07.01.04.05/2024). This study was based on secondary use of pseudonymized administrative data. In accordance with the Finnish Act on the Secondary Use of Health and Social Data (552/2019) and the research permit, individual patient consent or separate ethical review was not required. The study is compliant with applicable data protection regulations, including the EU General Data Protection Regulation (GDPR 2016/679).

## Supplementary information

Supplementary Information

## Data Availability

All data produced during this study are included in this published article or supplementary information. Due to data protection regulation, patient-level data cannot be shared.

## Code availability

The underlying code for this study is available at https://github.com/achdahlberg/cost-analysis-2025.git.
