# Supplementary material for: Cost minimization analysis of digital-first healthcare pathways in primary care
Source: NPJ Digit Med. 2025 Aug 25;8:546. doi: 10.1038/s41746-025-01937-z (PMC12379152; doi:10.1038/s41746-025-01937-z)
Supplement: Supplementary file 1 — Supplementary Information [file 41746_2025_1937_MOESM1_ESM.pdf]

Supplementary Information

Figures

Supplementary Figure 1. **Sankey diagram of digital-first pathway.** The diagram depicts patient progression through successive encounters in the digital-first pathway, beginning with a digital clinic contact. Node sizes reflect the proportion of patients at each step; flows represent transitions between encounter types. Percentages indicate the distribution at each stage. “Episode Ends” marks the absence of further recorded encounters.

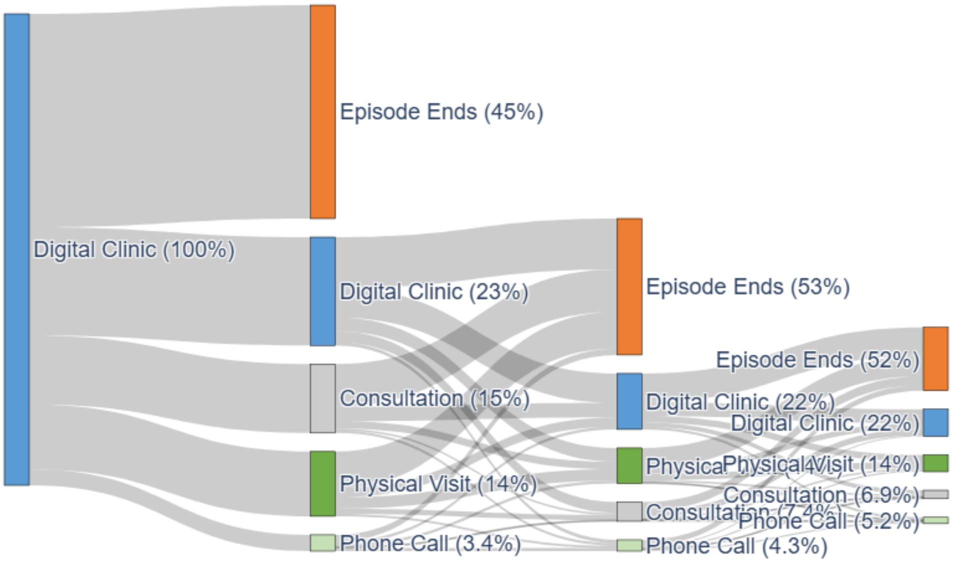

Supplementary Figure 2. **Sankey diagram of traditional pathway.** The diagram shows patient flow through successive encounters in the traditional pathway after propensity score matching, beginning with a physical visit or phone call. Node sizes reflect the proportion of patients at each step; flow widths represent transitions between encounter types. Percentages indicate the distribution at each stage. “Episode Ends” marks the absence of further recorded encounters.

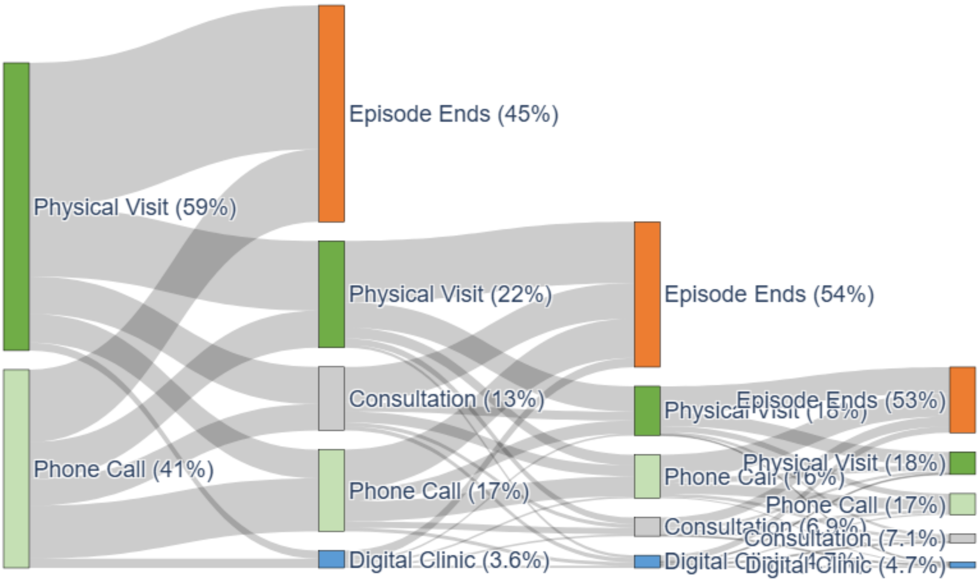

Supplementary Figure 3. Weekly pattern of return visits following episode start in primary healthcare. This figure illustrates the temporal pattern of healthcare utilization after the initiation of a care episode. We analyzed 8-week follow-up data from primary healthcare episodes with defined care pathways. Each episode was included if it had a complete 8-week follow-up period available in the dataset (i.e., it started at least 8 weeks before the end of the data collection period).

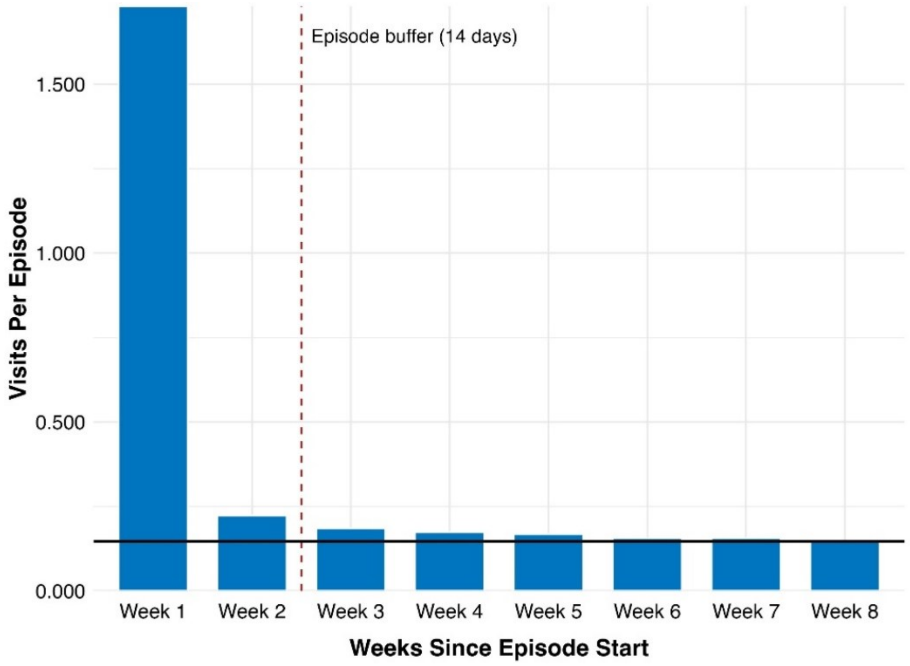

Tables

Supplementary Table 1. Breakdown of mean episode costs (€) by clinical presentation and healthcare pathway. Percentages indicate relative savings of the digital-first pathway compared to the traditional pathway.

Category	Digital-first pathway, mean per episode (€)	Traditional pathway, mean per episode (€)	*t*-statistic	*P*-value	Cost savings
Dermatological symptoms					
Encounter	152.18	227.56	-29.60	<.001	33.1%
Laboratory	2.13	4.25	-9.58	<.001	49.9%
Imaging	0.90	1.99	-5.20	<.001	54.8%
Gastroenteritis					
Encounter	122.12	244.86	-9.28	<.001	50.1%
Laboratory	1.41	14.70	-6.76	<.001	90.4%
Imaging	0.48	1.80	-1.85	.06	73.3%
Ophthalmological symptoms					
Encounter	166.61	246.52	-13.42	<.001	32.4%
Laboratory	1.35	2.19	-2.35	.02	38.4%
Imaging	0.42	1.89	-4.16	<.001	77.8%
Respiratory infections					
Encounter	175.90	192.88	-7.91	<.001	8.8%
Laboratory	3.32	6.02	-6.11	<.001	44.9%
Imaging	1.05	2.07	-7.48	<.001	49.3%
Urinary tract infections					
Encounter	183.38	232.91	-8.30	<.001	21.3%
Laboratory	11.38	19.72	-10.15	<.001	42.3%
Imaging	0.63	1.88	-3.62	<.001	66.5%

Supplementary Table 2. Sensitivity analysis: CMA results using unmatched patient groups. This table presents the results of a sensitivity analysis comparing mean total episode costs (€) between digital-first and traditional pathways without propensity score matching (unmatched analysis).

	Digital-first pathway, total costs (mean per episode, €)	Traditional pathway, total costs (mean per episode, €)	*t*-statistic	*P*-value	Cost savings
Dermatological symptoms	152.72	254.21	-48.30	<.001	39.9%
Gastroenteritis	130.95	283.17	-12.58	<.001	53.8%
Ophthalmological symptoms	158.92	257.19	-22.11	<.001	38.2%
Respiratory infections	180.27	218.56	-18.87	<.001	17.5%
Urinary tract infections	191.37	260.32	-12.27	<.001	26.1%

Supplementary Table 3. **Sensitivity analysis: CMA results using same patient.** This sensitivity analysis compares costs within the same patients who used both digital-first and traditional healthcare pathways, controlling patient-level variability.

	Digital-first pathway, mean total cost per episode (€)	Traditional pathway, mean total cost per episode (€)	N of pairs	*t*-statistic	*P*-value	Cost savings
Dermatological symptoms	237.94	329.50	1 223	-7.17	<.001	27.8%
Gastroenteritis	216.94	295.87	13	-1.12	.28	26.7%
Ophthalmological symptoms	212.13	278.90	116	-3.19	.002	23.9%
Respiratory infections	291.68	309.97	1 355	-1.86	.06	5.9%
Urinary tract infections	288.54	325.65	136	-1.13	.26	11.6%

Supplementary Table 4. Sensitivity analysis: CMA results using alternative unit costs (THL pricing catalog). Inflation-adjusted standardized national pricing data from the Finnish Institute for Health and Welfare (THL)[1,2].

	Digital-first pathway, mean total cost per episode (€)	Traditional pathway, mean total cost per episode (€)	*t*-statistic	*P*-value	N of pairs	Cost savings
Dermatological symptoms	80.15	127.13	-31.85	<.001	6 927	37.0%
Gastroenteritis	58.01	136.47	-10.86	<.001	312	57.5%
Ophthalmological symptoms	99.18	142.61	-12.37	<.001	1 461	30.5%
Respiratory infections	87.82	106.11	-14.49	<.001	9 616	17.2%
Urinary tract infections	108.06	139.54	-8.71	<.001	1 381	22.6%

Supplementary Table 5. Sensitivity analysis: CMA results for 7-day episode window.

	Digital-first pathway, mean total cost per episode (€)	Traditional pathway, mean total cost per episode (€)	*t*-statistic	*P*-value	N of pairs	Cost savings
Dermatological symptoms	122.27	204.36	-41.78	<.001	7 502	40.2%
Gastroenteritis	102.23	229.00	-12.29	<.001	355	55.4%
Ophthalmological symptoms	142.19	231.55	-19.59	<.001	1 766	38.6%
Respiratory infections	145.79	187.78	-22.43	<.001	9 321	22.4%
Urinary tract infections	162.63	225.67	-12.71	<.001	1 516	27.9%

Supplementary Table 6. Sensitivity analysis: CMA results for 30-day episode window.

	Digital-first pathway, mean total cost per episode (€)	Traditional pathway, mean total cost per episode (€)	*t*-statistic	*P*-value	N of pairs	Cost savings
Dermatological symptoms	180.68	285.67	-28.69	<.001	6 058	36.8%
Gastroenteritis	151.13	295.49	-9.23	<.001	299	48.9%
Ophthalmological symptoms	183.07	287.02	-15.16	<.001	1 562	36.2%
Respiratory infections	194.42	242.10	-15.66	<.001	7 506	19.7%
Urinary tract infections	224.54	303.38	-9.69	<.001	1 297	26.0%

Supplementary Table 7. Sensitivity analysis: Mean episode costs using inverse probability of treatment weighting (IPTW). Weighted mean total costs per episode calculated via inverse probability of treatment weighting (IPTW), an approach estimating the average treatment effect across the full study population. Weights were truncated at the 1st and 99th percentiles to mitigate extreme values.

	Weighted mean cost (€)
Dermatological symptoms	
Digital-first	169.97
Traditional	248.17
Gastroenteritis	
Digital-first	133.93
Traditional	267.07
Ophthalmological symptoms	
Digital-first	166.01
Traditional	245.53
Respiratory infections	
Digital-first	189.59
Traditional	212.49
Urinary tract infections	
Digital-first	194.61
Traditional	256.03

Supplementary Table 8. Sensitivity analysis: Weighted Least Squares (WLS) regression results (IPTW analysis). Weighted least squares regression was conducted as part of the IPTW sensitivity analysis to quantify cost differences between digital-first and traditional care, adjusting for propensity scores.

	Coefficient	Adjusted R^2	*t*-statistic	*P*-value	Cost savings
Dermatological symptoms	-78.20	0.050	-37.54	<.001	31.5%
Gastroenteritis	-133.14	0.131	-13.47	<.001	49.9%
Ophthalmological symptoms	-79.52	0.058	-18.53	<.001	32.4%
Respiratory infections	-22.90	0.005	-11.66	<.001	10.8%
Urinary tract infections	-61.42	0.032	-11.16	<.001	24.0%

Supplementary Table 9. Definition of medication categories based on ATC classification system. Categorized medications based on the Anatomical Therapeutic Chemical (ATC) classification system, which is maintained by the WHO Collaborating Centre for Drug Statistics Methodology.

Medication category	ATC code pattern	Definition	Common examples
Systematic antibiotics	J01	Antibacterials for systemic use	Amoxicillin, doxycycline, azithromycin, cephalexin
UTI-specific antibiotics	J01XE01, J01CA08, J01EA01	First-line antibiotics for urinary tract infections according to Finnish treatment guidelines	Nitrofurantoin, pivmecillinam, trimethoprim
Topical antimicrobials for skin	D01, D06	Antifungals and antibiotics for dermatological use	Clotrimazole, miconazole, fusidic acid, mupirocin
Topical antimicrobials for eye	S01A	Ophthalmological anti-infectives	Chloramphenicol, fusidic acid, ofloxacin eye drops
Opioid analgesics	N02A	Opioid pain medications	Codeine, tramadol, oxycodone
NSAIDs and paracetamol	N02B	Non-opioid analgesics and antipyretics	Ibuprofen, naproxen, paracetamol (acetaminophen)
Inhaled respiratory medications	R03A, R03B	Inhalants for obstructive airway diseases	Salbutamol, formoterol, beclomethasone, fluticasone inhalers

Supplementary Table 10. Prescription medication use in matched patient cohorts. Values are presented as percentages of patients who received each medication type during the 14-day episode. Differences were calculated as digital-first minus traditional pathway values. Statistical significance was determined using chi-square tests for binary outcomes (medication use vs. non-use).

Pathway	Medication	Digital-first (%)	Traditional (%)	Difference	*P*-value
Respiratory infection	Systemic antibiotics	12.6	20.9	-8.3	<.001
Respiratory infection	Opioid analgesics	0.7	1.1	-0.4	0.015
Respiratory infection	NSAIDs/paracetamol	2.0	2.8	-0.7	0.002
Respiratory infection	Respiratory medication	2.5	3.8	-1.3	<.001
Respiratory infection	Cough and cold medication	2.5	4.7	-2.2	<.001
Skin symptoms	Systemic antibiotics	12.1	16.6	-4.5	<.001
Skin symptoms	Topical skin medication	5.5	5.6	-0.1	0.876
Urinary tract infection	Systemic antibiotics	68.3	62.6	+5.8	0.005
Urinary tract infection	UTI antibiotics	65.4	56.3	+9.1	<.001
Eye symptoms ("acute")	Topical eye medication	61.5	56.2	+5.3	0.002
Gastroenteritis	Systemic antibiotics	2.6	5.7	-3.1	0.202

Supplementary Table 11. CHEERS 2022 Checklist[3]. Completed checklist outlining how each item in the CHEERS 2022 reporting guideline for health economic evaluations was addressed in the manuscript.

	Item	Guidance for Reporting	Reported in section
**TITLE**			
Title	1	Identify the study as an economic evaluation and specify the interventions being compared	1
**ABSTRACT**			
Abstract	2	Provide a structured summary that highlights context, key methods, results and alternative analyses.	1-2
**INTRODUCTION**			
Background and objectives	3	Give the context for the study, the study question and its practical relevance for decision making in policy or practice.	2-3
**METHODS**			
Health economic analysis plan	4	Indicate whether a health economic analysis plan was developed and where available.	Not applicable
Study population	5	Describe characteristics of the study population (such as age range, demographics, socioeconomic, or clinical characteristics).	15-16, Table 1 & 2
Setting and location	6	Provide relevant contextual information that may influence findings.	15-16
Comparators	7	Describe the interventions or strategies being compared and why chosen.	17-18
Perspective	8	State the perspective(s) adopted by the study and why chosen.	15
Time horizon	9	State the time horizon for the study and why appropriate.	18-19
Discount rate	10	Report the discount rate(s) and reason chosen.	Not applicable
Selection of outcomes	11	Describe what outcomes were used as the measure(s) of benefit(s) and harm(s).	20
Measurement of outcomes	12	Describe how outcomes used to capture benefit(s) and harm(s) were measured.	20
Valuation of outcomes	13	Describe the population and methods used to measure and value outcomes.	20-21
Measurement and valuation of resources and costs	14	Describe how costs were valued.	19-20
Currency, price date, and conversion	15	Report the dates of the estimated resource quantities and unit costs, plus the currency and year of conversion.	19
Rationale and description of model	16	If modelling is used, describe in detail and why used. Report if the model is publicly available and where it can be accessed.	20
Analytics and assumptions	17	Describe any methods for analysing or statistically transforming data, any extrapolation methods, and approaches for validating any model used.	6, 20-21
Characterizing heterogeneity	18	Describe any methods used for estimating how the results of the study vary for sub-groups.	6, Table 1 & 2
Characterizing distributional effects	19	Describe how impacts are distributed across different individuals or adjustments made to reflect priority populations.	Table 1 & 2
Characterizing uncertainty	20	Describe methods to characterize any sources of uncertainty in the analysis.	6, 21
Approach to engagement with	21	Describe any approaches to engage patients or service recipients, the general public, communities, or stakeholders (e.g., clinicians or payers) in the design of the study.	Not applicable

patients and others affected by the study			
**RESULTS**			
Study parameters	22	Report all analytic inputs (e.g., values, ranges, references) including uncertainty or distributional assumptions.	Table 1 & 2
Summary of main results	23	Report the mean values for the main categories of costs and outcomes of interest and summarise them in the most appropriate overall measure.	Table 3 & 4
Effect of uncertainty	24	Describe how uncertainty about analytic judgments, inputs, or projections affect findings. Report the effect of choice of discount rate and time horizon, if applicable.	11-14
Effect of engagement with patients and others affected by the study	25	Report on any difference patient/service recipient, general public, community, or stakeholder involvement made to the approach or findings of the study	Not applicable
**DISCUSSION**			
Study findings, limitations, generalizability, and current knowledge	26	Report key findings, limitations, ethical or equity considerations not captured, and how these could impact patients, policy, or practice.	6-15
**OTHER RELEVANT INFORMATION**			
Source of funding	27	Describe how the study was funded and any role of the funder in the identification, design, conduct, and reporting of the analysis	22-23
Conflicts of interest	28	Report authors conflicts of interest according to journal or International Committee of Medical Journal Editors requirements.	23

Supplementary Table 12. Diagnosis codes included in the study. The selected diagnoses represent the most frequently encountered acute conditions managed through the digital-first pathway, comprising approximately 50% of all encounters within this care model. These conditions were chosen based on their high prevalence, suitability for remote assessment, and clinical relevance for both digital-first and traditional care pathways. The inclusion of both ICD-10 and ICPC-2 codes ensures comprehensive classification and facilitates comparability across different healthcare coding systems.

Diagnosis codes	ICD-10	ICPC-2
Dermatological symptoms	L	S01-S76, S81-S99
Gastroenteritis	A08-A09	D73
Ophthalmological symptoms	H00, H01, H04.10, H10, H11.0, H11.3	F01, F02, F03, F13, F15, F16, F29, F70, F71, F72, F73, F75
Respiratory infections	J00-J22	R05, R08, R09, R21, R25, R71, R72, R74, R75, R76, R77, R78, R80, R83
Urinary tract infections	N30.0, N30.9	U01, U02, U05, U07, U13, U70, U71, U72

Supplementary Table 13. **Unit costs for healthcare encounters**. Encounter-specific costs (in euros) based on the publicly available service price list[4], confirmed by the Päijät-Häme Wellbeing Services County. These unit costs were applied to estimate total episode-level costs.

	Päijät-Häme
Nurses' digital appointment	€61
Nurses' appointment	€98
Doctors' consultation	€55
Doctors' digital appointment	€100
Doctors' physical appointment	€166

References

1 Finnish Institute for Health and Welfare. *Terveyden- ja sosiaalihuollon yksikkökustannukset Suomessa vuonna 2017*, <https://www.julkari.fi/bitstream/handle/10024/142882/URN_ISBN_978-952-343-493-6.pdf?sequence=1&isAllowed=y> (2021).

2 Official Statistics of Finland. *Consumer Price Index (2015=100), yearly data, 2015-2023 (11xc)*, <https://pxdata.stat.fi/PxWeb/pxweb/en/StatFin/StatFin__khi/statfin_khi_pxt_11xc.px/table/tableViewLayout1/> (2024).

3 Husereau D, Drummond M, Augustovski F, de Bekker-Grob E, Briggs AH, Carswell C, Caulley L, Chaiyakunapruk N, Greenberg D, Loder E, Mauskopf J, Mullins CD, Petrou S, Pwu RF, Staniszewska S; CHEERS 2022 ISPOR Good Research Practices Task Force. Consolidated Health Economic Evaluation Reporting Standards 2022 (CHEERS 2022) Statement: Updated Reporting Guidance for Health Economic Evaluations. BMJ. 2022;376:e067975.

4 Wellbeing Services County of Päijät-Häme. *Tuotehinnasto*, <https://www.paijatha.fi/wp-content/uploads/2024/01/Paijat-Hameen-hyvinvointialueen-tuotehinnasto-2024.pdf> (2024).
